# Occlusive retinal vasculitis and periphlebitis in Buerger’s disease: a case report

**DOI:** 10.1186/s12348-020-00222-2

**Published:** 2020-10-30

**Authors:** Ioannis S. Dimopoulos, Michael Dollin, Chloe C. Gottlieb

**Affiliations:** 1 Department of Ophthalmology, University of Ottawa Eye Institute, Ottawa, ON Canada; 2 The Ottawa Hospital Research Institute, General Campus, 501 Smyth Road, CCW, Box 307, Ottawa, ON K1H 8L6 Canada

## Introduction

Thromboangiitis obliterans (TAO), also known as Buerger’s disease, is a segmental occlusive nonatherosclerotic inflammatory condition of small and medium sized arteries and veins of the upper and lower extremities [1]. Leo Buerger originally described the condition in 1908 among young Polish and Russian Jews in New York city. Buerger’s originally proposed pathologic process involved the concept of a thromboarteritis or thrombophlebitis, rather than the proliferative or obliterating process derived from the intima of the arteries and veins (obliterating endarteritis.) Consensus today is that TAO is a different entity from closely related pathologic disorders, such as arteriosclerosis obliterans and thromboembolism and can involve other vascular territories including cerebral, coronary, renal and mesenteric arteries [2]. The etiology of this condition remains unknown, but tobacco is thought to play a critical role in its pathophysiology. For that reason, the diagnostic criteria for TAO includes positive smoking history, in addition to onset prior to 50 years of age, infra-popliteal arterial occlusive disease, upper limb involvement or phlebitis migrans, and the absence of atherosclerotic risk factors except smoking [3].

Limited published data exist on the ocular involvement in TAO. Bernardczykowa and Zawilski [4] reported fundoscopic findings in the spectrum of hypertensive retinopathy among 52 affected individuals, including narrowing of the retinal arteries, early sclerotic changes and arteriosclerosis. Case reports have also described other rare ocular manifestations of TAO, varying from non-arteritic ischemic optic neuropathy (NAION) [5, 6] and papillophlebitis [7] to normal tension glaucoma [8]. There has only been a single case report of central retinal artery ischemic event associated with TAO [9] that lacked visible retinal emboli. However, retinal vasculitis with evidence of perivascular inflammation and vessel wall stain/leakage has not been previously described in TAO. We herein report, for the first time, angiographic findings suggestive of occlusive retinal vasculitis and periphlebitis in an adult patient with presumed TAO.

## Case presentation

A 48-year-old male with a history of recurrent ulcers at the tip of his fingers for the past 3 years presented to his local ophthalmologist in North Bay, Ontario after noticing sudden onset vision loss in his right eye for 1 week. On exam, his visual acuity was recorded as 20/500 in the right eye and 20/20 in the left. Dilated fundus exam revealed an ischemic event of his right retina with cotton-wool spots but without any definite emboli. He was urgently sent to the emergency department for stroke workup. A CT scan of the brain was obtained, which showed no abnormalities. He was started on aspirin 81 mg po daily and subsequently referred to stroke prevention clinic and assessed by neurology. His blood work revealed a normal HbA1c and lipid panel. The only abnormality detected was mild macrocytosis, possibly secondary to alcohol intake. His carotid doppler scan was normal with no evidence of carotid artery stenosis. MRI imaging of the brain revealed only a small hyperintensity in the posterior cortex of the cerebellum that was confirmed to be an artifact on repeat single axial non 3-D FLAIR.

### Systemic investigations

Over the following months, he underwent extensive workup by internal medicine, dermatology, rheumatology and vascular surgery for his intermittent facial and upper extremity ulcerative lesions, which were worse in the winter time. He was also noted to have digital pitting and finger nail bed changes with dilated capillaries and dropout. Patient denied any Raynaud’s phenomena, with no noticeable skin colour changes when fingers were immersed in cold water. He also denied hematuria, renal disease, oral and genital ulcers. There was no previous history of scleritis, uveitis or symptoms suggestive of autoimmune disease. His social history was significant for cigarette smoking (½ - 1 pack per day; total of 20 pack/years) and daily alcohol intake (4–6 beers/day). There was no documented illicit drug use. The family history was significant for Raynaud’s disease, fibromyalgia and thyroid disease in his maternal aunt, and prostate cancer in his father.

On exam, no carotid, iliac, renal or femoral bruits, palpable purpura or tophaceous changes were appreciated. Blood pressure measurements showed no inter-arm difference or elevated systolic or diastolic pressure (100/80 right and 102/78 left, respectively). Laboratory investigations were significant only for decreased activated protein C and functional antithrombin III. Histologic analysis of his facial lesions revealed dermal telangiectasias, raising clinical suspicion of scleroderma. However, all immunological tests for rheumatic and vasculitic diseases were negative, including antinuclear antibody testing (× 3), anti-neutrophilic cytoplasmic autoantibodies (ANCA) and scleroderma-specific antibodies. A summary of all laboratory investigations conducted is provided in Table 1. Furthermore, computed tomography (CT) of the chest and abdomen with contrast showed no specific features of scleroderma or elements of CREST syndrome. Pulmonary functions tests were interpreted as normal with no evidence of chronic obstructive pulmonary disease (COPD) or restrictive lung disease. Upper limb arterial duplex scan showed abnormal photo-plethysomography in the upper extremity digits indicating possible small vessel occlusive disease and mild degree of reverse flow in the left ulnar artery. Repeat carotid doppler ultrasonography demonstrated normal peak systolic velocities with no evidence of any significant degree of carotid artery stenosis.

**Table 1:** Laboratory Investigations

	Value/Result
Hematology	
Hemoglobin	154 g/L
ESR	7 mm/hr
C-Reactive Protein	1.6 mg/dl
Biochemistry	
Hemoglobin A1c	5.3%
Low-density Lipoprotein (LDL)	2.9 mmol/L
Creatinine	66 μmol/L
GFR	> 60 ml/min/1.73m^2^
aPTT	26 s
INR	1.1
Homocysteine	Normal
Immunology	
Antinuclear antibodies (ANA)	Negative
Rheumatoid factor (RF)	Negative
Anti-CCP antibodies	Negative
Anti-ENA antibodies	Negative
Complement C3	1.2 g/L
Complement C4	0.12 g/L
Beta-2-glycoprotein antibodies	Negative
ANCA	Negative
Antiglobulin test	Negative
Smooth muscle antibodies (SMA)	Negative
Anti-cardiolipin antibodies	Negative
Anti-centromere antibodies (ACA)	Negative
Anti-Scl-70 antibodies	Negative
Hypercoagulable Panel	
Free protein S	0.79 U/mL
Total protein S	0.86 U/mL
Activated protein C	0.56 U/mL **
Functional antithrombin III	0.65 U/mL **
Factor V Leiden	Negative
Factor 20210A	Negative
Lupus anticoagulant	Negative

*ESR* erythrocyte sedimentation rate, *GFR* glomerular filtration rate, *aPTT* activated partial thromboplastin time, *INR* international normalized ratio, *CCP* cyclic citrullinated peptide, *ENA* extractable nuclear antigen, *ANCA* anti-neutrophil cytoplasmic antibody; **: denotes abnormal values

In light of all scleroderma/systemic sclerosis investigations being negative, a presumptive diagnosis of thromboangiitis obliterans with coagulation disorder (protein C and antithrombin III deficiency) was entertained. Patient was counselled on smoking cessation with the aid of varenicline and started on antihypertensive therapy amlopidine 2.5 mg po daily.

### Second eye involvement

A few months later and 1 year after the initial retinal ischemic event, the patient experienced sudden-onset severe vision loss in his left eye. He was assessed by his local ophthalmologist who noted a similar picture of ischemic retinopathy in the left eye without definite emboli. Patient was urgently referred to our tertiary eye care center in Ottawa for further evaluation. In view of his pro-thrombotic state and second retinal ischemic event, apixaban 5 mg po BID was prescribed by his internist and amlodipine was switched to nifedipine 30 mg po daily as an antihypertensive to enhance peripheral vascular blood flow. No prednisone was started given still the diagnostic concern over scleroderma and scleroderma-induced renal crisis.

**Fig. 1:**
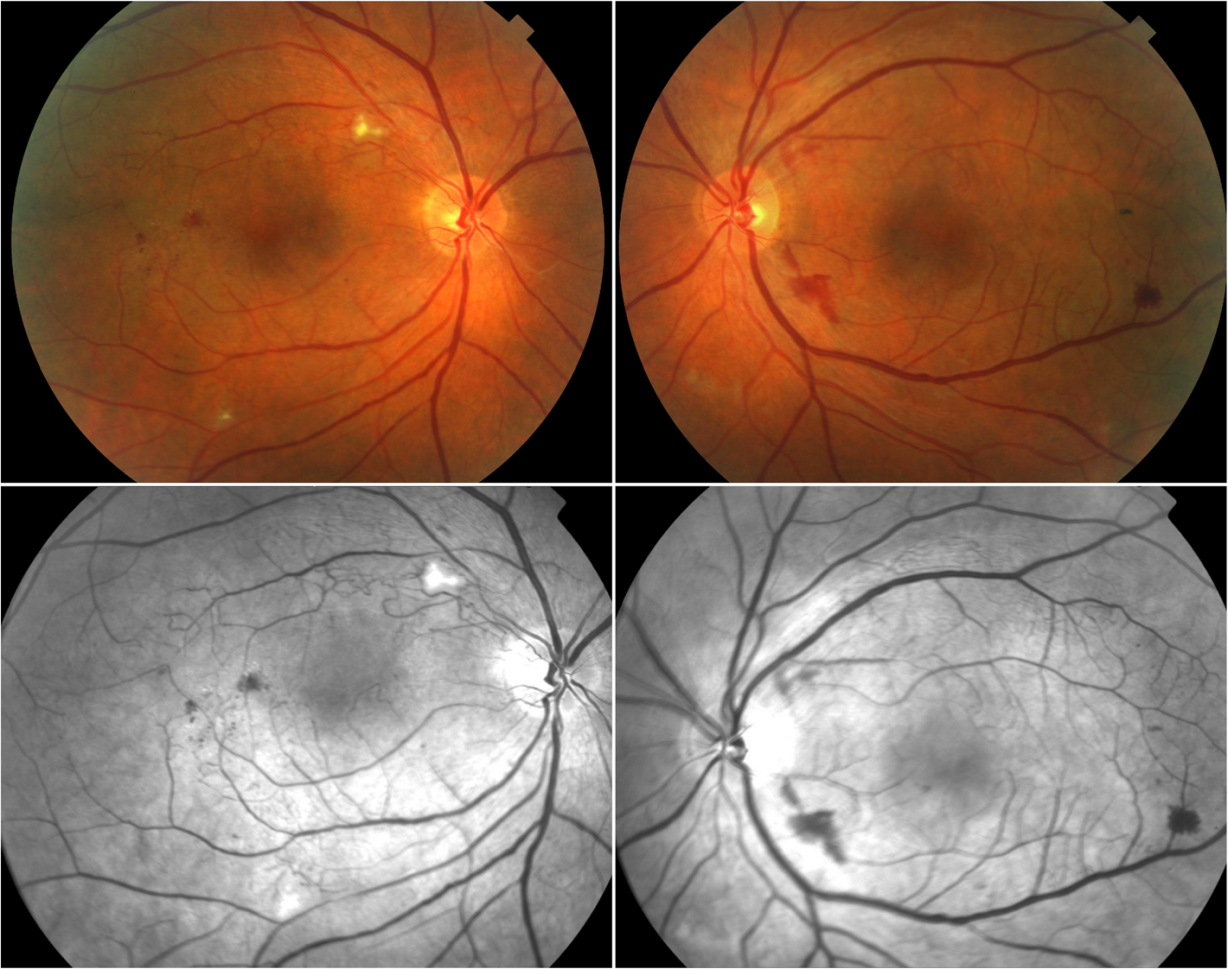
Colour fundus photos (*top row*) and corresponding red-free images (*bottom row*). Occlusive retinopathy in the macular region, with vascular loops, collateralization, nerve fiber layer infarcts and pre−/intra-retinal hemorrhages is noted. The vessels show arteriolar narrowing with venular dilation and sheathing

When examined by our service, visual acuity was counting fingers at 1 m in the right eye and 20/600 in the left eye. Intraocular pressures were 16 mmHg in both eyes. Slit lamp examination revealed no inflammation in the anterior chamber. Mild nuclear sclerotic cataract was present in both eyes. Dilated fundus examination showed significant retinal thinning in the macular region. Vascular loops were present with collateralization. A few nerve fiber layer infarcts were evident in the right eye along areas of occlusive retinopathy. Dot-blot and flame-shaped hemorrhages were noted in both eyes. The vessels showed arteriolar narrowing with venular dilation and sheathing (Fig. 1).

### Ocular imaging

**Fig. 2:**
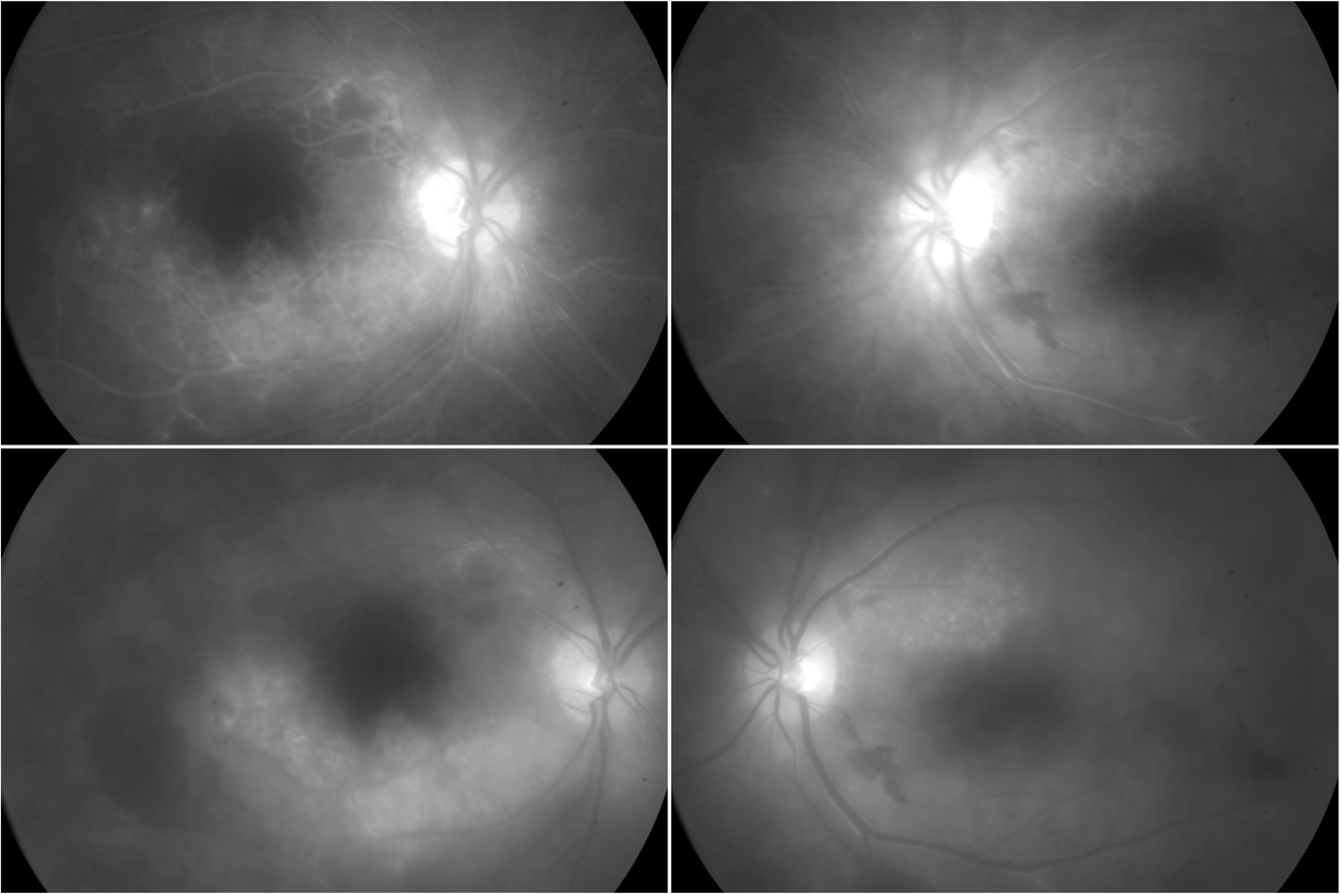
Fluorescein angiography in early (*top row*) and mid/late phases (*bottom row*). Early phase shows vessel wall staining involving veins and collateral arteries. Mid/late phase shows corresponding leakage. The FAZ appears enlarged greater in the right compared to left eye

**Fig. 3:**
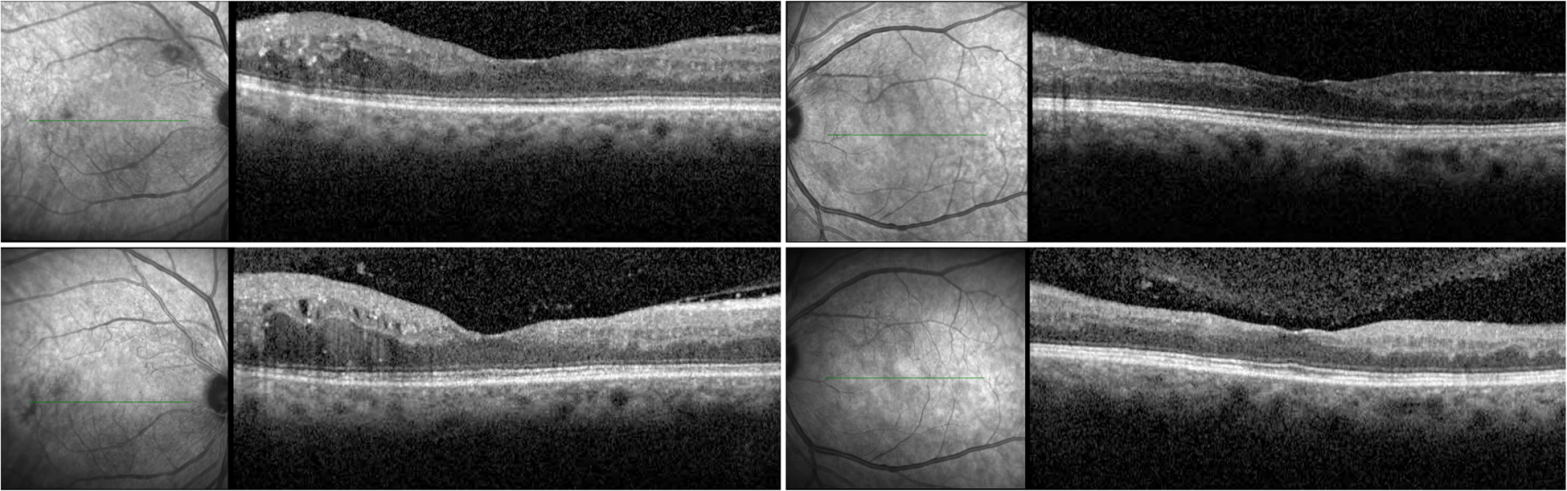
SD-OCT horizontal transfoveal B-scans of the right and left eye at initial assessment (*top row*) and 3 months later (*bottom row*). Inner retinal atrophy with paracentral cystoid degeneration and edema is demonstrated in both eyes

Fluorescein angiography revealed early phase vessel wall staining involving veins and collateral arteries and mid/late phase leakage. The arm-retinal and arteriovenous transit times for the left eye were recorded as 16.8 s and 11.3 s, respectively. The foveal avascular zone (FAZ) was enlarged more in the right eye compared to left (Fig. 2). Spectral domain optical coherence tomography (Spectralis OCT; Heidelberg Engineering, Heidelberg, Germany) imaging revealed areas of inner retinal atrophy with paracentral cystoid degeneration and edema in both eyes (Fig. 3). Follow-up OCT imaging 3 months later showed no change in retinal thickness or cystoid degeneration morphology.

## Discussion

TAO is known to be a segmental occlusive inflammatory condition of small and medium sized arteries and veins of the upper and lower extremities. Retinal vasculitis has not been clinically and/or angiographically described in the spectrum of TAO. However, various studies have shown that TAO is a generalized functional arterial disorder, with impaired endothelial function related to increased levels of various inflammatory markers [10]. Recent immunohistochemistry studies have demonstrated that T-cell mediated immune inflammation is the main pathogenetic mechanism in the development of TAO [11]. CD4+ cells are found predominantly in the inflammatory infiltrate of TAO- affected vessels and adventitia, in addition to CD20, CD31, CD68, adhesion molecules and interferon-gamma [11]. Anti-endothelial and anti-neutrophil cytoplasmic antibodies have also been described in correlation with disease activity in patients with TAO [12].

A recent publication suggested that the spectrum of TAO disease is gradually changing, with a male-to-female ratio that is decreasing (3:1), shift in the age of diagnosis to older age and more upper-extremity involvement [13]. Nevertheless, knowledge about ocular involvement appears limited. In the most extensive case series by Bernardczykowa and Zawilski [4], vascular changes caused by arteriosclerosis and thrombotic occlusions are known to occur within the retinal arteries in TAO patients with stenotic arteritis or arteriosclerotic stenosis of the lower extremities.

In our case, the presence of co-existent coagulation abnormalities caused a diagnostic dilemma. It is plausible that the prothrombotic effect of smoking in conjunction with the underlying coagulation abnormalities could have contributed to the clinical presentation. Over 30 patients have been described in the literature with retinal vascular occlusions associated with underlying abnormalities in the hemostatic system [14]. However, the majority of them seem to preferentially affect the retinal venous system (vein occlusions). There have been limited reports of retinal arterial occlusions in protein C and familial antithrombin III deficiency [15–17]. Usually, the presence of antiphospholipid antibodies is the most frequently reported coagulopathy associated with arterial occlusions [14]. In some TAO afflicted patients, the presence of antiphospholipid antibodies and coagulation abnormalities, such as hyperhomocysteinemia, protein S and protein C deficiencies, has been identified [18]. However, no clear evidence of a coagulation abnormality exists in TAO.

TAO remains an independent clinicopathologic entity with definite diagnosis dependent upon histopathological criteria. Clinically, the distal nature of disease pattern and upper limb involvement are key discriminatory features. Confident clinical diagnosis should be made when a set of 5 diagnostic criteria has been fully met, although this recommendation is not universally accepted [3]. In our case, 4/5 diagnostic criteria of TAO were met (smoking history, onset before the age of 50 years, upper limb involvement, absence of atherosclerotic risk factors other than smoking). In addition, colour duplex ultrasound and photoplethysmographic waveform analysis supported small vessel occlusive disease in the upper extremity digits with normal proximal vasculature. Helpful angiographic findings of collateralization that have been previously described in TAO (“corkscrew,” “spider legs,” or “tree roots”) were not present, but these are not pathognomonic or necessarily required for diagnosis [3].

In conclusion, retinal vasculitis with evidence of perivascular inflammation and vessel wall stain/leakage on angiography has not been previously described in TAO. We herein expand the retinal vascular phenotype of the disease to include occlusive retinal vasculitis and periphlebitis. TAO should be considered in the investigation of non-infectious retinal vasculitis in the proper clinical context.

## Data Availability

Not applicable.

## Abbreviations

TAO: Thromboangiitis obliterans
SD-OCT: Spectral-domain optical coherence tomography
FAZ: Foveal avascular zone
